# Electric-field control of spin accumulation direction for spin-orbit torques

**DOI:** 10.1038/s41467-018-08274-8

**Published:** 2019-01-16

**Authors:** Rahul Mishra, Farzad Mahfouzi, Dushyant Kumar, Kaiming Cai, Mengji Chen, Xuepeng Qiu, Nicholas Kioussis, Hyunsoo Yang

**Affiliations:** 1 Department of Electrical and Computer Engineering, National University of Singapore, Singapore, 117576 Singapore; 2 Department of Physics and Astronomy, California State University, Northridge, CA 91330-8268 USA; 3 Shanghai Key Laboratory of Special Artificial Microstructure Materials & School of Physics Science and Engineering, Tongji University, Shanghai, 200092 China

## Abstract

Electric field is an energy-efficient tool that can be leveraged to control spin–orbit torques (SOTs). Although the amount of current-induced spin accumulation in a heavy metal (HM)/ferromagnet (FM) heterostructure can be regulated to a certain degree using an electric field in various materials, the control of its direction has remained elusive so far. Here, we report that both the direction and amount of current-induced spin accumulation at the HM/FM interface can be dynamically controlled using an electric field in an oxide capped SOT device. The applied electric field transports oxygen ions and modulates the HM/FM interfacial chemistry resulting in an interplay between the spin Hall and the interfacial torques which in turn facilitates a non-volatile and reversible control over the direction and magnitude of SOTs. Our electric-field controlled spin-orbitronics device can be programmed to behave either like the SOT systems with a positive spin Hall angle or a negative spin Hall angle.

## Abstract (web summary)

Voltage control of spin-orbit torques (SOTs) provides additional degrees of freedom for spinmemory and -logic applications. Here the authors demonstrate a large voltage control of direction as well as amount of current-induced spin accumulation at the heavy metal/ferromagnet interface and effectively tune the SOTs in a Pt/Co/GdOx heterostructure.

## Introduction

In a HM/FM heterostructure, current-induced spin accumulation^1–5^ at the HM/FM interface arises from the bulk spin Hall effect (SHE)^2,6–9^ and/or from the interfacial spin–orbit coupling^1,6–9^. The direction of this spin accumulation is an important parameter in the design of SOT applications such as the magnetic random access memory (MRAM), domain wall memory and skyrmion-based memory as it determines the direction of magnetization switching, domain wall motion and skyrmion motion. However, the relative direction between the current and the generated spin accumulation for a given heterostructure is always fixed which inhibits a flexible design of the above applications. For example, a fixed direction of spin accumulation necessitates the change in the polarity of the applied current in order to switch between two magnetic states^2,4^ which will result in an additional circuitry in SOT-MRAM^10,11^. In addition, selective switching of multiple bits sharing the same HM write layer^12^ cannot be achieved with a fixed and identical direction of spin accumulation. In view of the above drawbacks and requirements, it is imperative to be able to control the direction of current-induced spin accumulation in SOT devices.

In the previous works, the direction of SOTs was altered through stack engineering which involved methods such as a substitution of the spin Hall source^2,13^, variation of the heavy metal^6,14,15^ or capping layer thickness^9^ and usage of a dual heavy metal layers^16^. However, all the above methods for the control of SOT direction involve physical alteration of the heterostructure which is not feasible once the SOT devices have been fabricated. Here, we report a modulation of the SOT direction in a Pt/Co/GdO_*x*_ heterostructure by changing the concentration of oxygen in the Co layer using an electric field. The direction of spin accumulation is tuned reversibly in a non-volatile fashion to be in either the $\hat i \times \hat n$ or $- \hat i \times \hat n$ direction in a single device. Here, $\hat i$ and $\hat n$ represent the direction of the current flow and the film normal, respectively. In essence, our devices can be controlled to behave to be possessing either a positive or a negative effective spin Hall angle without any physical stack modifications. While the previous works^17–21^ have offered only a limited electric-field control on the magnitude of SOTs, we demonstrate a sizable control over both the magnitude as well as direction of SOTs. Our findings will offer a substantial improvement to the SOT based memory scheme as well as create opportunities for novel applications such as spin-logic circuits and programmable spin-circuits^22^.

## Results

### Harmonic measurements to evaluate the SOT polarity

**Fig. 1:**
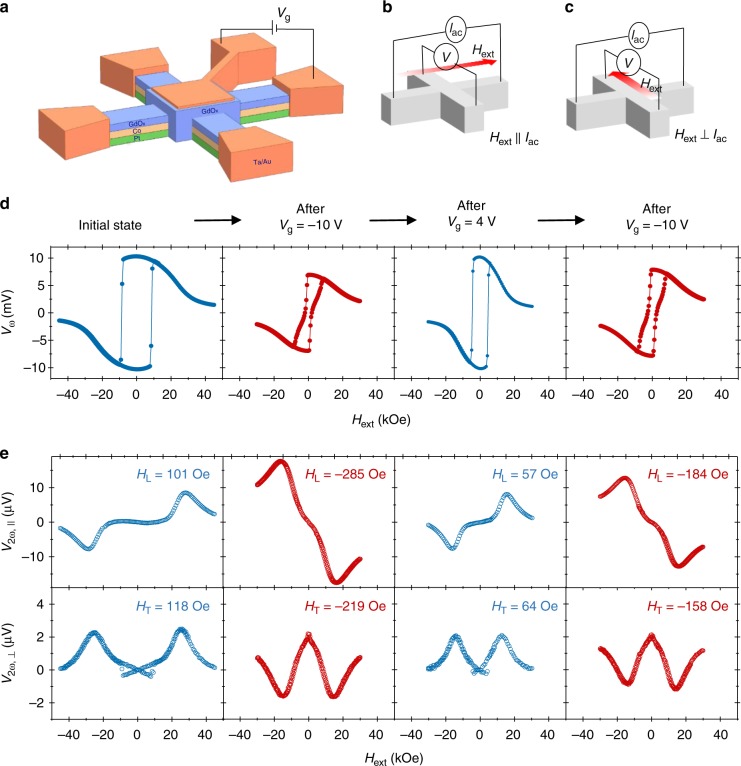
Effect of gate voltage application on the SOT effective fields. **a** Schematic of the Hall bar used for the electrical measurements. **b**, **c** *V*_*ω*,2*ω*_ are obtained with *H*_ext_ applied parallel (||) and perpendicular (⊥) to the ac current (*I*_ac_) flow direction. The measurement schematic with *H*_ext_ || *I*_ac_ (**b**) and *H*_ext_ ⊥ *I*_ac_ (**c**) are also shown. **d** *V*_*ω*_ and **e** *V*_2*ω*_ signals for a Pt (2 nm)/Co (0.8 nm) device as a function of in-plane external magnetic field (*H*_ext_), with subsequent gate voltage (*V*_g_) application events. Measurements were performed at 150 K. At this temperature, the SOT device displayed a good PMA in both the normal- and reversed-states. Both the normal- and reversed-states of devices persist at room temperature as well. The values of *H*_L_ and *H*_T_ corresponding to a current density of 10^12^ A m^−2^ are also shown in **e**. The planar Hall effect was considered in evaluating the SOT effective fields and the anomalous Nernst effect contribution was subtracted from the raw second harmonic data before plotting it

For the experiments, film stacks with the structure of substrate/MgO/Pt/Co/GdO_*x*_ were deposited on a Si/SiO_2_ substrate. These stacks were then patterned into Hall bar devices with a top GdO_*x*_ gate oxide as shown in Fig. 1a. In order to evaluate the direction and magnitude of the SOTs, the harmonic Hall measurement technique was employed^6,7,23,24^, in which an ac current (*I*_ac_) is passed through the Hall channel in presence of an in-plane magnetic field (*H*_ext_). The second harmonic measurements in *H*_ext_ || *I*_ac_ (Fig. 1b) and *H*_ext_ ⊥ *I*_ac_ (Fig. 1c) configurations primarily provide information of the longitudinal (*H*_L_) and the transverse (*H*_T_) SOT effective field, respectively. The first (*V*_*ω*_) and second harmonic (*V*_2*ω*_) Hall voltages for a Pt (2 nm)/Co (0.8 nm)/GdO_*x*_ (70 nm) device are shown in Fig. 1d, e, respectively. For the case of initial device state before application of any gate voltage (*V*_g_), the sign of the second harmonic peaks (*SH*_peaks_) in Fig. 1e is consistent with the SOT direction from a typical Pt underlayer^7^, corresponding to a positive effective spin Hall angle or SOT efficiency, *ξ*_SOT_ due to the damping-like torque. This state of the device is referred to as the “normal state”. *H*_L_ and *H*_T_ are 101 and 118 Oe per 10^12^ A m^−2^, respectively, for the initial normal state.

Subsequently, a *V*_g_ of −10 V was applied for 120 s on the top gate electrode at 40 °C as illustrated in Fig. 1a. An elevated temperature was used for efficient oxygen migration^25–27^. Harmonic measurements were then performed after setting *V*_g_ = 0 V and removing the gate voltage connections. The first harmonic hysteresis loop has the same polarity as compared to the initial device state as shown in Fig. 1d. However, the signs of *SH*_peaks_ for both *H*_ext_ || *I*_ac_ and *H*_ext_ ⊥ *I*_ac_ configurations are opposite in comparison with the normal device, as shown in Fig. 1e. This reversal of the *SH*_peaks_ indicates that both the *H*_L_ and *H*_T_, and hence the spin accumulation in the device, change their directions after negative *V*_g_ application. Therefore, the SOT direction becomes “negative” rendering the device into the “reversed state” (corresponding to a negative *ξ*_SOT_). The values of *H*_L_ and *H*_T_ in this reversed state are −285 and −219 Oe per 10^12^ A m^−2^, respectively. A *V*_g_ of +4 V was then applied on the reversed device for 120 s. Subsequent harmonic measurements show that the sign of *SH*_peaks_, and hence the SOT directions, return to their normal state (Fig. 1e). The SOT direction was again reversed by applying a *V*_g_ of −10 V for 120 s. Therefore, we observe that the direction of the current-induced spin accumulation and the sign of *ξ*_SOT_ can be reversibly toggled in the device through gate voltage applications. Moreover, both the normal- and reversed-states of the device are non-volatile and can only be modified by the gate voltage.

### Current-dependence for the normal- and reversed device

**Fig. 2:**
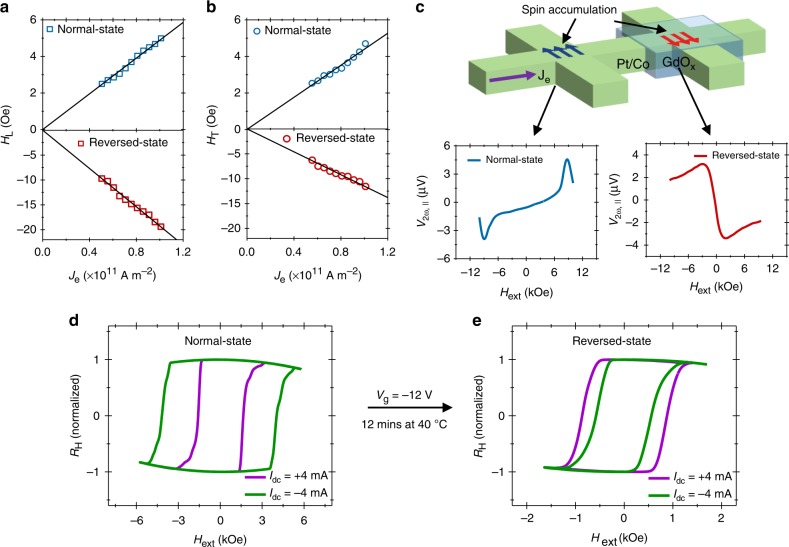
Current-dependence of effective fields and programmable spin accumulation. **a**, **b** Longitudinal (**a**) and transverse (**b**) effective fields (*H*_L_ and *H*_T_) as a function of current density (*J*_e_) for a Pt (2 nm)/Co (0.8 nm) Hall bar device. The solid lines are the linear fits. Measurements were done at 150 K. **c** Schematic of a two-bit Hall bar with one of the bit covered by gate oxide. Bottom figures show the second harmonic signals measured at room temperature in different regions of 2-bit Hall bar. The red and blue arrows in the schematic represent the current-induced spin accumulation directions. **d**, **e** Normalized Hall resistance (*R*_H_) versus in-plane external magnetic field (*H*_ext_) in the presence of a dc current (*I*_dc_) for a Pt (2 nm)/Co (0.8 nm) device in the normal state (**d**) and after reversing its state (**e**). Measurements were performed at 250 K at which the reversed device regained PMA

Figure 2a, b shows that the extracted values of the SOT effective fields have a linear dependence on the current magnitude for both the normal and reversed device states, thereby confirming their current-based origin. In addition, we show that different polarities of *ξ*_SOT_ can be programmed at different locations on a single-device channel (Fig. 2c). A 2-bit Hall bar was fabricated with one of the bits covered with a gate oxide as shown in Fig. 2c. The direction of spin accumulation of the gated-bit is programmed by applying a negative *V*_g_ to be opposite to that of the ungated bit as can be seen from the second harmonic signals measured with *H*_ext_ || *I*_ac_ shown in Fig. 2c.

### Magnetic hysteresis loop measurement to evaluate the SOT polarity

In order to further verify the observed reversal of SOTs, the anomalous Hall effect (AHE) was measured with positive and negative dc currents (±4 mA) and with *H*_ext_ applied in the direction of currents with a tilt of ~2° towards the film normal^2^. For our measurement configuration, the coercivity of the AHE loop should decrease (increase) for a normal state, when a positive (negative) dc current is applied. This is indeed what we observe for a device in the normal state, as shown in Fig. 2d. Conversely, when a negative *V*_g_ was applied to reverse the state of the device, the coercivity of the AHE loop with a positive dc current is larger compared to that with a negative dc current (Fig. 2e). This observation further confirms the opposite direction of the SOTs in the reversed device. It should be noted that the sign reversal of SOT is accompanied by a reduction in *R*_AHE_ and perpendicular magnetic anisotropy (PMA), *H*_k_, of the devices due to Co oxidation (Supplementary Fig. 1). For the device shown in Fig. 2d, e the *R*_AHE_ and *H*_k_ reduce from 2.9 Ω and 10.6 kOe to 1.7 Ω and 4.2 kOe from a normal to reversed state, respectively. The non-volatile and reversal modification of the SOT direction was also confirmed on multiple devices (Supplementary Fig. 2). In addition, the sign reversal of SOT can be achieved for a Co layer as thick as ~2 nm, thereby signifying the feasibility of realizing a thermally stable SOT memory element (Supplementary Fig. 3). Moreover, by reducing the gate oxide thickness the SOT directions can be toggled in the order of seconds at room temperature (Supplementary Fig. 2).

### Mechanism of the electric-field control

**Fig. 3:**
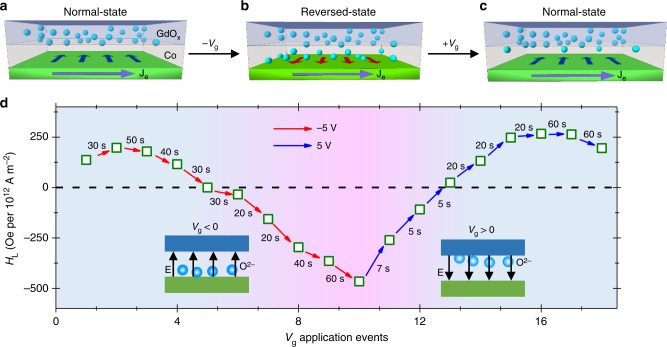
Evolution of the device state. **a**–**c** Schematic showing a Pt/Co/GdO_*x*_ device in a normal (**a**, **c**) and reversed state (**b**). The oxygen (blue sphere) at the Co/GdO_*x*_ interface in a normal device moves to the Co/Pt interface on application of negative gate voltage and gives rise to an interfacial torque. The direction of spin accumulation in **a**–**c** is indicated by the blue and red arrows. **d** Evolution of *H*_L_ in a Pt (1.5 nm)/Co (0.8 nm)/GdO_*x*_ (20 nm) device on application of gate voltage pulses. *V*_g_ pulses were applied at room temperature while the *H*_L_ measurements were performed at 160 K after removing the *V*_g_ connections. Red arrows represent applications of negative *V*_g_ pulse while blue arrows indicate positive *V*_g_ application events. The duration of the *V*_g_ pulse is indicated adjacent to the arrows

The origin of the voltage control of the SOT direction can be explained by the oxygen ion (O^2−^) migration in the presence of an electric field. In a normal device as shown in Fig. 3a, the SOTs are mainly induced by the SHE of Pt^7,8^. Although the broken inversion symmetry is also a source of interfacial SOTs like the Rashba torque^8,9,28^, its magnitude is smaller than that of SHE^8^. Upon application of a negative *V*_g_, the oxygen is driven from the ionic-conductive GdO_*x*_ gate into the Co layer as shown in Fig. 3b. Previously, ionic migration in GdO_*x*_ has been used to modulate the anisotropy landscape of underlying magnet^25,26^ and later to completely quench its magnetization^27^. It should be noted that apart from the electric-field induced migration of oxygen ion, another competing mechanism for Co oxidation/reduction which involves hydrolysis of water has also been proposed recently^29^. Nevertheless, when the Pt/Co interface is oxidized, it modifies the polarity and strength of the Rashba SOTs^9,30–32^ by modulating the Rashba coefficient (*α*_R_), which is sensitive to the spin–orbit coupling strength and the band structure^31,33^, which in turn depend on the interfacial composition and quality. A similar modification of polarity and magnitude of *α*_R_ with oxidation has been predicted and observed earlier on magnetic metallic surfaces^31^. With progressive oxidation of the Co layer and hence the Pt/Co interface, the magnitude of this interfacial Rashba SOT whose polarity is opposite to that of SHE, keeps increasing and when it becomes larger than the SHE-based SOT, a sign reversal of SOTs is realized. It should be noted that oxygen ions cannot migrate beyond the Pt/Co interface into the Pt layer due to the high redox potential of Pt. This is also confirmed by applying negative *V*_g_ on a Pt/GdO_*x*_ device which results in no change in the device resistance (Supplementary Fig. 5). When a positive *V*_g_ is applied on a reversed device, the O^2−^ at the Pt/Co interface are driven back into the GdO_*x*_ layer as shown in Fig. 3c. In this case, the device returns to the normal state with the observed SOTs being dominated by the SHE. Since the oxygen can be driven back and forth from the Pt/Co interface by *V*_g_ applications, the state of the device is both reversible and non-volatile.

The underlying competition between the interfacial Rashba and the spin Hall SOTs was further examined by performing a Pt thickness-dependent study (Supplementary Note 5). We find that while devices with a thin Pt layer (< 2.5 nm) showed SOT sign reversals on application of gate voltages, no such effect was observed for thicker Pt samples (≥ 2.5 nm) (Supplementary Fig. 6). A relatively small spin Hall angle^34,35^ and a large interface (Pt/Co) to bulk (Pt) ratio in a thin Pt-based heterostructure results in the dominance of the interfacial SOTs over the spin Hall torque after sufficient Pt/Co interfacial oxidation. Hence, a thin Pt device easily enters the reversed state compared to a thick (≥ 2.5 nm) Pt device. For devices with a thicker Pt layer, the magnitude of the Rashba torque never dominates over the SHE torque and the SOT device always remains in the normal state. We also perform the Co thickness dependence study in order to obtain additional insight into the role of oxygen in the Co layer (Supplementary Note 3). It is expected that a large Co thickness should result in larger screening effect thereby making O^2−^ migration in the Co and subsequent SOT reversal challenging. Indeed, we find that the temperature or thermal energy required to modulate SOTs increases with the increasing the Co thickness (Supplementary Fig. 4). We also ensure that the Co/GdO_*x*_ interface is not the source of reversed SOT. For this a Co (1 nm)/GdO_*x*_ heterostructure was deposited without any Pt layer. There is no visible second harmonic signal for this device (Supplementary Fig. 8).

In order to gain additional insight into the progressive evolution of SOTs in the devices, *H*_L_ was measured for a Pt (1.5 nm)/Co (0.8 nm) device after application of successive *V*_g_ pulses at room temperature. As shown in Fig. 3d, negative *V*_g_ pulse applications, after starting from a normal device state with *H*_L_ of 136 Oe per 10^12^ A m^−2^, reduce the *H*_L_ slowly before quenching it to zero. The maximum *ξ*_SOT_ (Supplementary Fig. 9) evaluated for the normal device is 0.05. With each subsequent negative *V*_g_ pulses, the *H*_L_ gradually evolves in the negative direction due to progressive oxidation of the Pt/Co interface which results in build-up of Rashba SOTs of negative polarity. We find the minimum ratio of oxygen over cobalt in the Co layer for the sign reversal of SOT to be ~0.35 (Supplementary Fig. 10). The reversed device shows a maximum negative *H*_L_ value of −465 Oe per 10^12^ A m^−2^ which corresponds to *ξ*_SOT_ *=* −0.10. Consequently, when positive *V*_g_ pulses were applied on this reversed device, the magnitude of *H*_L_ begins to reduce as the oxygen starts to migrate away from the Pt/Co interface thereby reducing the interfacial SOTs. The *H*_L_ is quenched to zero before finally returning to the normal state as shown in Fig. 3d. The progressive evolution from a normal to a reversed state and back to the normal state articulates the competition of the interfacial and spin Hall SOTs depending on the amount of Pt/Co interfacial oxidation. Furthermore, the above results demonstrate that a desired magnitude and direction of SOTs can be programmed in the device with *ξ*_SOT_ ranging from 0.05 to −0.10.

### First principle calculations

**Fig. 4:**
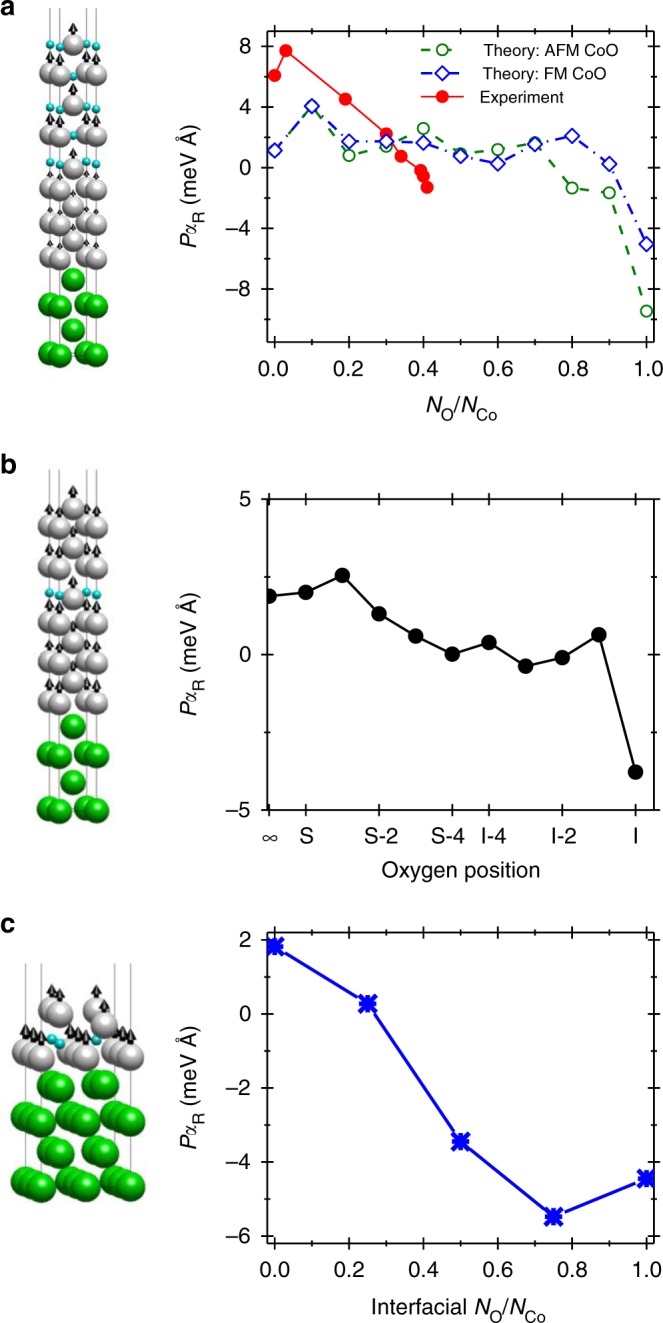
Rashba spin–orbit coupling calculations. **a** Effective Rashba coefficient (ERC), *Pα*_R_, as a function of oxygen concentration, *x* = *N*_O_/*N*_Co_, in the Pt (4 ML)/Co (10(1−*x*) ML)/CoO (10*x* ML) slab for both ferromagnetic (FM) and antiferromagnetic (AFM) ordering of CoO. A representative atomic structure used for calculations is shown on the left side of the graph (CoO (5 ML)/Co (5 ML)/Pt (4 ML) with FM ordering). The green, silver and blue sphere represent the Pt, Co and O atoms, respectively. Experimental values of ERC are also plotted for comparison. The calculations were carried out within the relaxation time approximation with a relatively large value of energy broadening, *η* *=* 0.1 eV, in order to emulate the interfacial roughness of the bilayer. **b** The calculated value of ERC versus the position of a single oxygen atom which is placed on different Co layers. The result at *x*→∞ denotes the position of the oxygen far away from the Co film, ‘S’ denotes the oxygen atom placed at the top Co layer while ‘I’ denotes oxygen placement at the Pt/Co interface. The representative atom structure on the left of **b** shows the Pt/Co structure with the oxygen atom placed at the S-4 layer. **c** Calculated ERC versus interfacial oxygen concentration for a Pt (4 ML)/Co (2 ML) bilayer where the ground state of CoO is ferromagnetic and *η* *=* 0.1 eV. The atomic structure for the (2 × 2) Pt (4 ML)/Co (2 ML) bilayer with interfacial *N*_O_/*N*_Co_ = 0.75 is shown on the left of **c**

In order to further elucidate the effect of Co oxidation on the SOTs in the Co/Pt bilayer we have carried out electronic structure calculations^36,37^ to evaluate the evolution of Rashba torque under different cases of oxidation (Supplementary Note 9). Figure 4a displays the variation of the effective Rashba coefficient (ERC) as a function of oxygen concentration, *x* = *N*_O_/*N*_Co_, in the Pt (4 ML)/Co (10(1−*x*) ML)/CoO (10*x* ML) slab (ML represents monolayer). Effectively, *x* = 0.1 means that oxygen is present only in the top layer while *x* = 1 represents completely oxidized Co with oxygen at the Pt/Co interface. The ERC, *Pα*_R_, was calculated from the field-like SOT ($\tau _{{\mathrm{FL}}}^0$), using the expression *Pα*_*R*_ = ħ^2^*M*_s_ τ_FL_^0^/2 *m*_e_*σ*_xx_^38^, where *P* is the polarization, *ħ* is the reduced Plank’s constant, *M*_s_ is the saturation magnetization, *m*_e_ is the free electron’s mass and *σ*_xx_ is the longitudinal conductivity of the structure. The theoretical calculations show an enhancement of the ERC when oxygen is placed on the top Co layer (Fig. 4a). This is in agreement with the experimental data presented in Fig. 3d in which an increase of *ξ*_SOT_ (Supplementary Fig. 9) was observed during initial oxidation. It should be noted that similar increase of *ξ*_SOT_ was observed in earlier works in which there was limited Co oxidation^17,32^. Furthermore, the calculations show a sign reversal of the ERC around *x* ≈ 0.8 which is the cause of the reversed SOT. It should be noted that in the calculations *x* > 0.8 corresponds to the presence of oxygen near the Co/Pt interface. Also note that the theoretical calculations show that the ERC is relatively insensitive to the type of magnetic ordering (ferromagnetic or antiferromagnetic) of the CoO layer.

In Fig. 4a we have also plotted the ERC extracted from the experimental data using the expression *Pα*_*R*_ = *μ*_B_*M*_s_*H*_T_/*J*_e_^39^, where *µ*_B_ is Bohr’s magneton. The experimental value of ERC also becomes negative upon increasing the oxygen concentration in the Co layer which corresponds to the SOT reversal. However, the value of *x* for which the sign reversal of ERC is observed in the experiment and the calculations is different due to the different way of oxygen filling for the two cases. While in the calculations the Co layers were completely oxidized one by one starting from the top, in the experiments the oxygen penetrates to the Pt/Co interface even before the top Co layers are completely oxidized. Since CoO is an insulator, increasing CoO thickness leads to the decrease of the effective Co thickness. In order to verify that the sign reversal of ERC is not due to the decrease in the effective Co thickness (Supplementary Fig. 11b), we have carried out first principles calculations with a single oxygen atom placed on the different Co layers. Figure 4b shows the ERC versus the position of the oxygen atom. The ERC shows an abrupt sign reversal when oxygen is placed at the Co/Pt interface.

A separate calculation using a Pt (4 ML)/Co (2 ML) slab with (2 × 2) planar supercell (compared to the (1 × 1) planar cell in Fig. 4a) was performed in order to quantify the interfacial oxygen concentration required to reverse the ERC or SOT. The larger planar area allows for the introduction of 4 oxygen atoms at the interface (only one oxygen atom per layer can be added in the (1 × 1) planar cell in Fig. 4a). We find that for oxygen concentration ~0.3, the ERC reverse its sign and its magnitude keeps on increasing with additional oxygen as shown in Fig. 4c. The structural geometry in this calculation and the subsequent result are in close agreement with the experiments for which an ERC sign reversal is obtained for *x* ~ 0.35 (Supplementary Fig. 10).

### Reversal of current-induced switching loop polarity

**Fig. 5:**
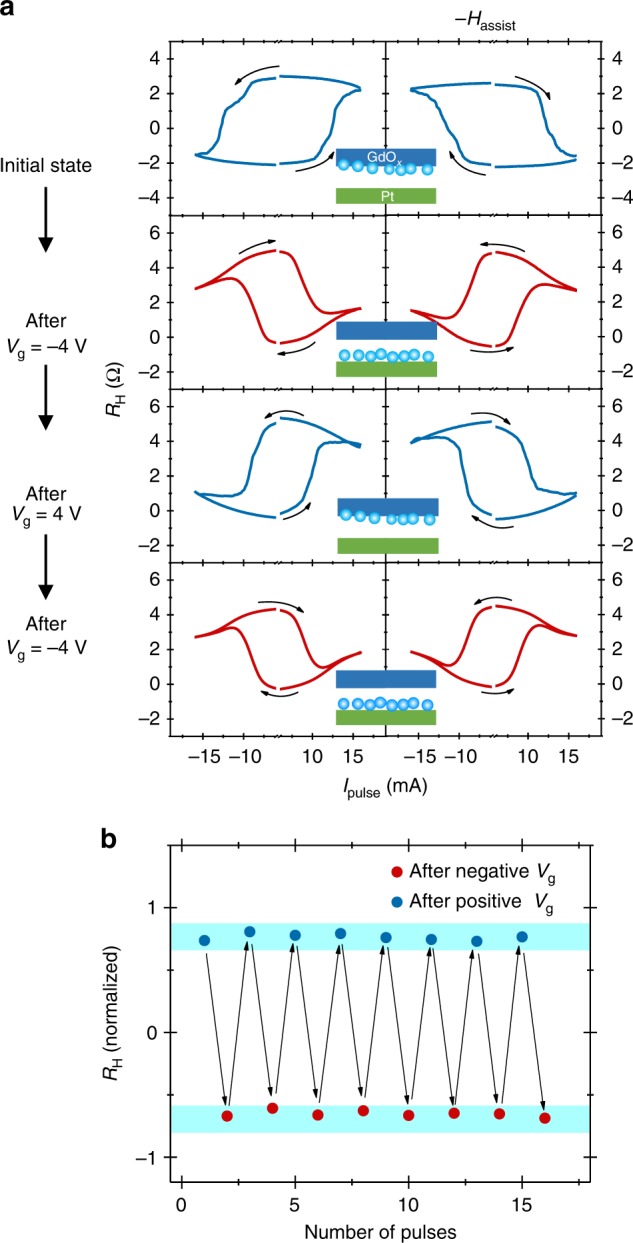
Current-induced switching measurements. **a** The Hall resistance (*R*_H_) as a function of pulses current magnitude (*I*_pulse_) for a Pt (1.5 nm)/Co (0.8 nm) device after successive *V*_*g*_ application at room temperature. The switching loops in **a** are shown for the assist field (*H*_assist_) direction along (+*H*_assist_) and opposite (−*H*_assist_) to the current flow direction. **b** Repeated toggling of the *R*_H_ by a unidirectional current after modulating the state of device with positive and negative *V*_g_. The switching measurements for the reversed device in **a**, **b** were performed at 150 K at which the device regained PMA

Finally, we demonstrate opposite current-induced magnetization switching sequences for the normal and the reversed device. Figure 5a shows the switching loops for the different device states for positive (left column) and negative (right column) directions of the assist field (*H*_assist_). The initial magnetization switching sequence for a normal device of Pt (1.5 nm)/Co (0.8 nm) is anti-clockwise (clockwise) for a positive (negative) direction of *H*_assist_. A *V*_g_ = −4 V for 120 s was then applied at room temperature to reverse the SOT directions. The subsequent switching measurement on this reversed device shows an opposite switching sequence that is clockwise (anti-clockwise) for the positive (negative) direction of *H*_assist_ (Fig. 5a). After subsequent application of a positive *V*_g_ for 60 s, the switching sequence reverts back to normal. Finally, the switching sequence is reversed again after the application of a negative *V*_g_. In essence, the device shows a unipolar switching behavior. For a given direction of the current and the *H*_assist_, the magnet can be stabilized in either upward or downward direction depending on the state of the device. The unipolar operation of the device, in which the device can switch between two states without changing the current polarity, is demonstrated in Fig. 5b using gating.

## Discussion

The modulation of SOTs is realized by regulating the amount of oxygen at the Pt/Co interface using an electric field. The reversible and non-volatile modulation (Supplementary Fig. 12) of SOT direction is demonstrated by both harmonic Hall voltage and current-induced switching measurements. The capability to generate an opposite switching sequence in a single device is a significant step toward a flexible realization of SOT-MRAM architecture. For example, simultaneous writing into different directions of multiple bits by placing them on a shared write layer served by a single transistor is possible. The control of SOT direction in a single device will not only accelerate the adoption of SOTs in spintronic memories but will also facilitate novel applications such as multi-bit memory cells, spin-logic devices and programmable spin-circuits.

## Methods

### Sample preparation

MgO (2 nm)/Pt (*t* nm)/Co (0.8 nm)/GdO_*x*_ (3 nm) films were deposited on a Si/SiO_2_ substrate using dc and rf magnetron sputtering at a base pressure of ~10^−9^ Torr. The Pt thickness, *t*, was varied from 1.5 to 3 nm. Thickness calibration was done prior to deposition. GdO_*x*_ for the top layer and the gate oxide was deposited using reactive sputtering of Gd in the presence of oxygen. An optimized oxygen flow rate of 0.5 sccm compared to 20 sccm for Argon was used during GdO_*x*_ deposition. Deposition pressure was kept less than 3 mTorr for all the layers. The as-deposited films were annealed at 250 °C for 1 h to induce PMA. The Hall channels were patterned using either photolithography or electron-beam lithography depending on their dimensions. This was followed by low power Ar-ion etching in an ion-milling chamber. Next, a top gate window was pattered to deposit GdO_*x*_ gate oxide. The gate oxide thickness was kept between 20 and 70 nm for different devices. In the final step, Ta (4 nm)/Au (70 nm) electrodes were deposited using either a sputter or an electron-beam evaporator. It should be noted that the thickness and quality of gate oxide determines the temperature and time duration for which the gate voltage is applied during different measurements. In addition, there was a device to device variation which is common in an oxide-based memristive systems^40^ and this affected our choice of gate voltage stimulus.

### Second harmonic measurements

A low frequency (13.7 Hz) ac current was passed through one of the Hall bar channels. The magnetic field was applied either along (*H*_ext_ || *I*_ac_) or orthogonal (*H*_ext_ ⊥ *I*_ac_) to the current flow direction (tilted ~2–4° to the sample normal). The first and second harmonic Hall voltages were recorded simultaneously while sweeping the magnetic field. For SOT effective field evaluation, a uniform current density was assumed through the heterostructure.

### Current-induced switching measurements

The anomalous Hall resistance was probed while sweeping a pulsed dc current through the Hall channel. 100 µs wide current pulses were used for the measurements. An in-plane assist field (*H*_assist_) of 1000 Oe was applied during the measurement to achieve deterministic switching. It should be noted that the device state (both normal and reversed) persists at room temperature, even though some experiments were conducted at low temperatures in order to regain PMA.

### First principles calculations

The density functional theory calculations for the Co/Pt bilayer slabs were carried out using the Vienna ab initio simulation package (VASP)^41,42^. The pseudopotential and wave functions are treated within the projector-augmented wave (PAW) method^43,44^. Structural relaxations were carried using the generalized gradient approximation as parameterized by Perdew et al.^45^ when the largest atomic force is smaller than 0.01 eV Å^−1^. A 15 Å thick vacuum region is introduced to separate the periodic slabs along the stacking direction. The plane wave cutoff energy is 500 eV and a 10 × 0 × 1 k-points mesh is used in the 2D Brillouin Zone (BZ) sampling. The GGA + U method with a Hubbard U of 3 eV has been used to treat the localized 3*d* orbitals of the Co atoms in the CoO monolayers (MLs). The tight-binding (TB) Hamiltonian was obtained from the VASP-Wannier90 calculations^36,46^, and the SOT was calculated using the approach introduced in ref. ^37^.

## Supplementary information

Supplementary Information

## Data Availability

The data that support the results of this study are available from the corresponding author upon reasonable request.
