# Supplementary material for: Electric-field control of spin accumulation direction for spin-orbit torques
Source: Nat Commun. 2019 Jan 16;10:248. doi: 10.1038/s41467-018-08274-8 (PMC6335414; doi:10.1038/s41467-018-08274-8)
Supplement: Supplementary file 1 — Supplementary Information [file 41467_2018_8274_MOESM1_ESM.pdf]

Electric-field control of spin accumulation direction for spin-orbit torques

Mishra *et al.*

Supplementary Note 1. Evolution of anomalous Hall resistance and perpendicular magnetic anisotropy with oxygen migration

While we have established that the direction of current-induced spin accumulation changes as oxygen penetrates to the Pt/Co interface. It should also be noted that other magnetic properties of the Pt/Co bilayer system which depend on interfacial and bulk oxygen in the Co are also modulated. In Fig. 3d of the main text, on applying negative gate voltage (V_g) to the Pt (1.5 nm)/Co (0.8 nm) device, oxygen migrates into the Co layer. This migration of oxygen in the Co layer leads to oxidation of the Co and formation of CoO which results in the reduction of the saturation magnetization (M_s) and anomalous Hall resistance (R_{AHE}). In Supplementary Fig. 1a we plot the value of R_{AHE} for the various device state, as a series of negative and positive V_g are applied on the device. On application of negative V_g, the R_{AHE} decreases continuously till a sign change of spin-orbit torques (SOT) is achieved. For the device under consideration, the value of R_{AHE} can be reduced by ~ 28 % compared to the initial value when measured at 160 K. The value of R_{AHE} can be regained again on applying a positive V_g to reduce the CoO into Co.

Apart from the modulation of R_{AHE}, the perpendicular magnetic anisotropy field (H_k) also undergoes drastic modulation under the application of negative V_g. The modulation in H_k is due to the change of the interfacial stoichiometry. In fact, for our samples the reversal of SOT is observed once the anisotropy of the sample turns in-plane at room temperature. As shown in Supplementary Fig. 1b the H_k reduces by ~ 33 % on application of negative V_g when measured at 160 K. Similar to the R_{AHE}, the H_k can also be regained back to its initial value by applying a positive V_g. It should however be noted that application of excess positive V_g makes the top Co interface devoid of oxygen and can again lead to a decrease of H_k as can be seen in the last two data points of the figure.

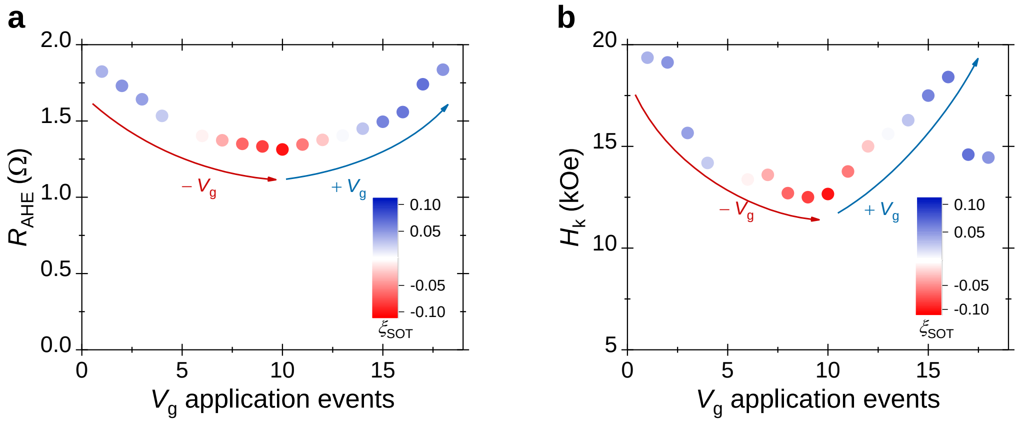

Supplementary Fig. 1. Progressive evolution of R_{AHE} and H_k. a,b, Evolution of R_{AHE} **(a)** and H_k **(b)** in a Pt (1.5 nm)/Co (0.8 nm) device on application of subsequent gate voltage pulses. The device state corresponds to that represented in Fig. 3d. The colour of data point represent the value of SOT efficiency (ξ_{SOT}). Measurements were conducted at 160 K.

Supplementary Note 2. SOT direction reversal in an 800 nm-wide device.

Supplementary Fig. 2 demonstrates the sign reversal of SOTs in an 800 nm-wide Hall channel device fabricated using electron beam lithography from a Pt (1.5 nm)/Co (0.8 nm) film. First (V_{ω}) and second harmonic ($V_{2\omega}$) signals measured for the initial state of the device are shown in top panel the figure. The sign of second harmonic signal for the device in its initial state is consistent with the normal sign of spin Hall angle for a Pt device. A negative V_g of −10 V was then applied on the device for 40 s at room temperature. Subsequent harmonic measurements reveal that the sign of second harmonic signal is opposite compared to the initial device state. This sign reversal of second harmonic signal signifies the underlying reversal in the direction of current-induced spin accumulation or the SOT polarity. The sign of spin accumulation or the SOT direction reverses again on applying a positive V_g of 4 V for 30 s. The bottom panel of the figure shows that the sign of second harmonic signal has returned to its initial state. It should be noted that for this device the modulations can be achieved by applying V_g only for few seconds at room temperature due to a use of thin gate oxide (20-30 nm). The successful demonstration of sign reversal of SOTs in a nanoscale device indicates the scalability of the proposed concept.

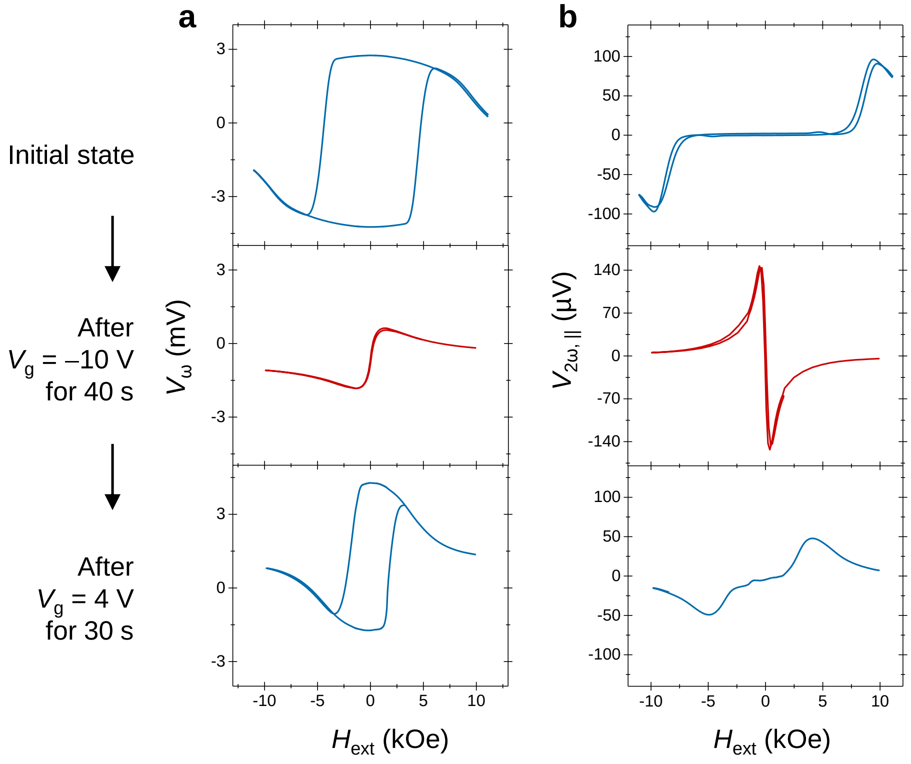

Supplementary Fig. 2. SOT direction reversal for an 800 nm-wide device. a,b, First **(a)** and second harmonic **(b)** measurement with H_{ext} || I_{ac} for an 800 nm-wide Pt (1.5 nm)/Co (0.8 nm) device with successive gate voltage application events at room temperature. The external field H_{ext} is the applied near-in-plane and I_{ac} is the ac current.

Supplementary Note 3. Co thickness dependence

Pt (1.5 nm)/Co (t_{Co} nm)/GdO_x samples were deposited and fabricated into gated Hall bar devices similar to the one shown in Fig. 1a of the main text. The Co thickness, t_{Co}, was varied from 0.8 to 2.5 nm and a ~ 30 nm thick GdO_x gate oxide was deposited on all the devices. In order to evaluate the polarity of SOT or current-induced spin accumulation in these devices, harmonic Hall measurements with $H_{ext} \parallel I_{ac}$ were performed (similar to the top panel of Fig. 1e). There are two main observations. First, the state of the device can be reversibly toggled in a non-volatile way for all the samples till $t_{Co} = 2$ nm. Second, the temperature at which V_g was applied in order to induce the SOT modulation increases with increasing the Co thickness.

Supplementary Fig. 3 shows the measured first and second harmonic signal ($H_{ext} \parallel I_{ac}$) for the Pt/Co samples with varying the Co thickness. The thickness of Co is indicated adjacent to each sub-figures. The state of all the devices reverses on application of negative V_g and subsequently when a positive V_g is applied the devices return to their normal state. The observed reversible and non-volatile modification of SOTs even for the devices with Co thickness of 2 nm signifies the feasibility of realizing a thermally stable SOT memory element. However, it should be noted that the temperature at which V_g was applied keeps on increasing with the Co thickness as shown in Supplementary Fig. 4a. This is primarily because the ionic migration inside Co becomes increasingly difficult with increasing the Co thickness due to the growing screening effect. Therefore, for a thicker Co layer a high temperature is required in order to provide enough energy for oxygen ions to migrate till the Pt/Co interface and induce SOT reversal. Since the temperature of our measurement setup was limited to 127 °C, we could not efficiently migrate the oxygen ions and induce SOT reversal in sample with $t_{Co} > 2$nm. For example, for sample with $t_{Co} = 2.2$ nm, the application of negative V_g at 127 °C for 8 hours did not result in any SOT reversal.

In order to show that increasing the Co thickness makes the oxygen migration challenging, a negative V_g of −3.5 V was applied at 100 °C on all the devices ($t_{Co} = 0.8$ to 2.3 nm) for 2400 s. The percentage change of R_{AHE} (ΔR_{AHE} %) as a function of Co thickness is plotted in Supplementary Fig. 4b. ΔR_{AHE} decreases with increasing the Co thickness signifying that under similar thermal and V_g stimulation, the ionic migration is largest for the thinnest device. For example, the ΔR_{AHE} is 73 % for t_{Co}= 0.8 nm, whereas it is mere 0.15 % for t_{Co}= 2.2 nm.

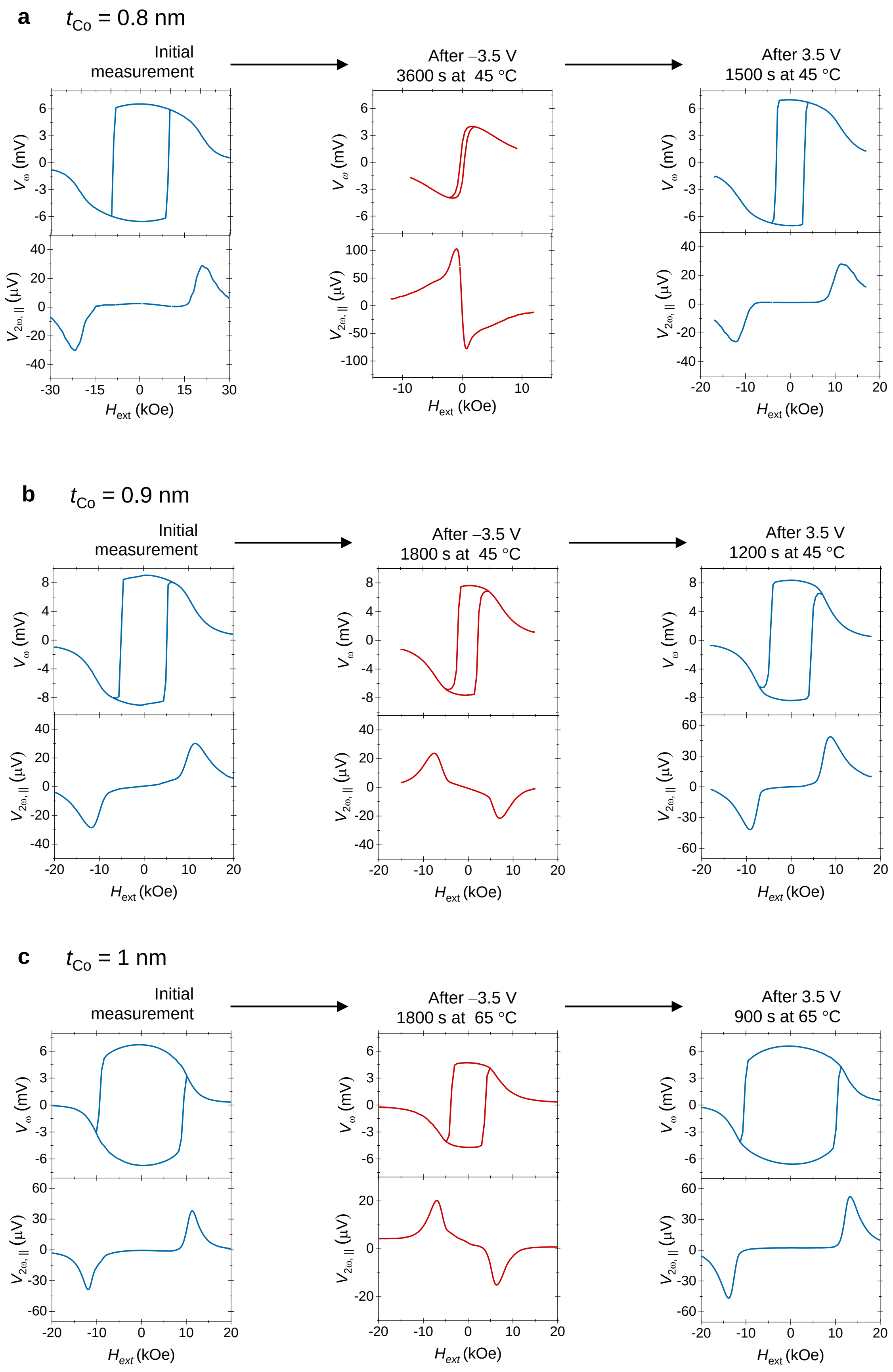

a
t_{Co} = 0.8 nm
Initial measurement
After −3.5 V 3600 s at 45 °C
After 3.5 V 1500 s at 45 °C
b
t_{Co} = 0.9 nm
Initial measurement
After −3.5 V 1800 s at 45 °C
After 3.5 V 1200 s at 45 °C
c
t_{Co} = 1 nm
Initial measurement
After −3.5 V 1800 s at 65 °C
After 3.5 V 900 s at 65 °C
V_{ω} (mV)
$V_{2\omega,\parallel}$ (μV)
H_{ext} (kOe)

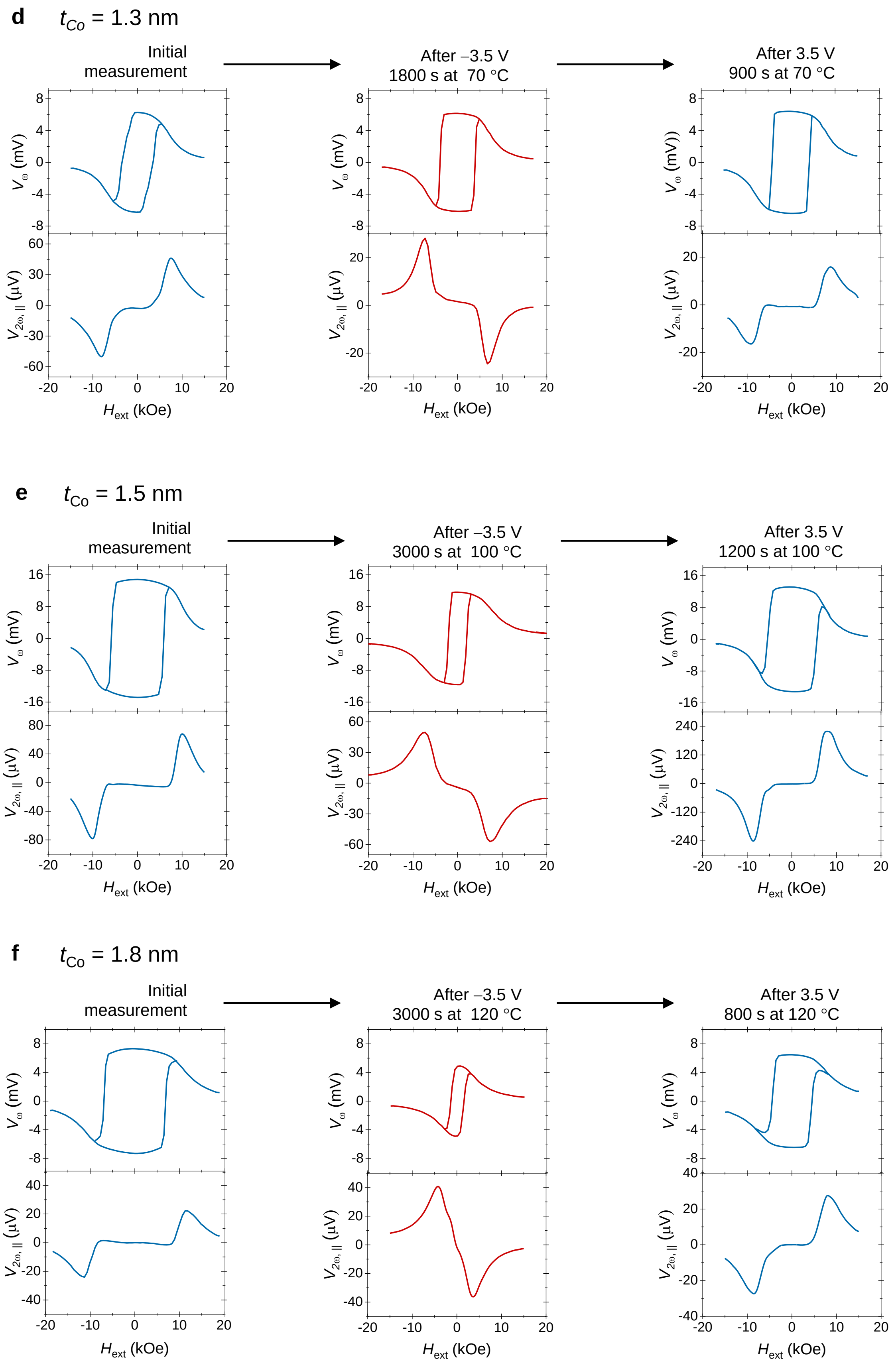
d
t_{Co} = 1.3 nm
Initial measurement
After –3.5 V 1800 s at 70 °C
After 3.5 V 900 s at 70 °C
e
t_{Co} = 1.5 nm
Initial measurement
After –3.5 V 3000 s at 100 °C
After 3.5 V 1200 s at 100 °C
f
t_{Co} = 1.8 nm
Initial measurement
After –3.5 V 3000 s at 120 °C
After 3.5 V 800 s at 120 °C
V_{ω} (mV)
$V_{2\omega,\parallel}$ (μV)
H_{ext} (kOe)

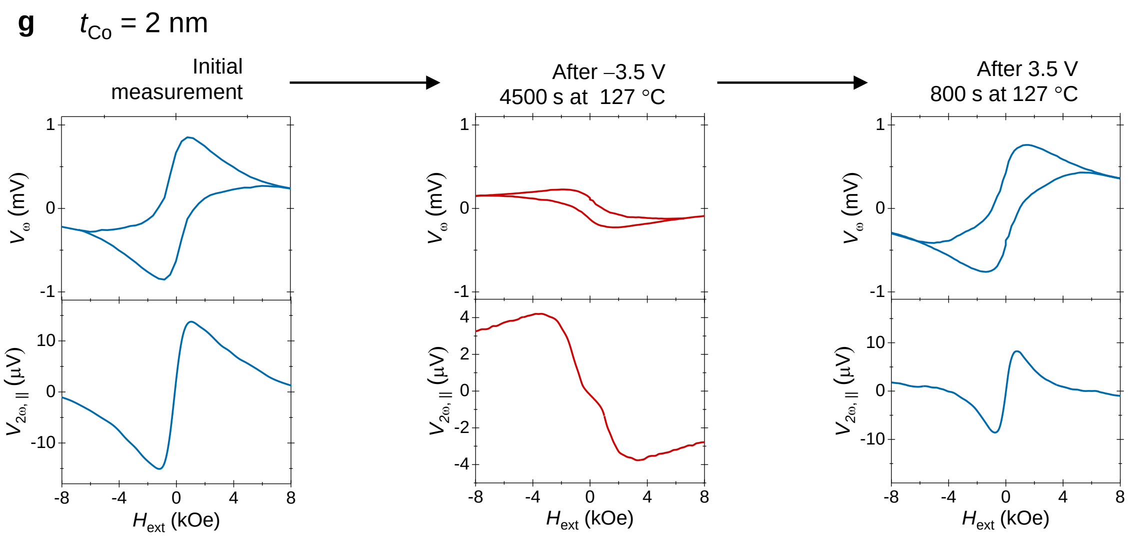

Supplementary Fig. 3. Modulation of SOTs with varying the Co thickness. a-g, First (V_ω) and second ($V_{2\omega}$) harmonic Hall signal for Pt (1.5 nm)/Co (t_{Co} nm)/GdO_x devices after gate voltage application events. The measurements were performed in the $H_{ext} \parallel I_{ac}$ configuration. Measurement temperature was kept at 225 K in order to ensure perpendicular anisotropy for the maximum number of devices.

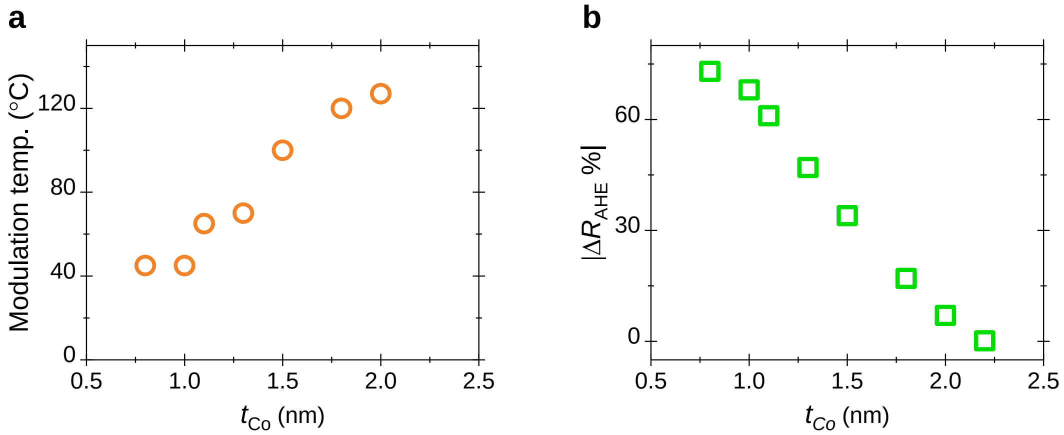

Supplementary Fig. 4. Modulation temperature and modulated R_{AHE} as a function of the Co thickness (t_{Co}). a, Temperature required to modulate the polarity of SOT as a function of Co thickness for a Pt (1.5 nm)/Co (t_{Co} nm) heterostructure. **b**, Percentage modulation of R_{AHE} as a function of Co thickness for a Pt (1.5 nm)/Co (t_{Co} nm) heterostructure. A V_g of −3.5 V was applied on all the devices for 40 minutes at 100 °C.

Supplementary Note 4. Oxidation of the Pt and Co layers

When a negative gate voltage (V_g) is applied on the device, the oxygen ions (O^{2-}) from the GdO_x penetrate into the Co layer and migrate towards the Pt/Co interface. However, because Pt is a noble metal, it is difficult for the O^{2-} to migrate into Pt and oxidize it. The redox potential of Pt is +1.18 V which indicates that it very difficult to oxidize Pt. In comparison, the redox potential of Co is −0.28 V, therefore it is easier to be oxidized. In order to confirm that O^{2-} does not penetrate into the Pt layer, two different sample stacks of MgO/Pt (1.5 nm)/GdO_x and MgO/Co (1 nm)/GdO_x were deposited and fabricated similar to that shown in Fig. 1a of the main text. The oxidation of Pt or Co can be confirmed by monitoring the device resistance after applying negative V_g, as oxidation would result in an increase of the device resistance. For example, the resistance of a Pt (2 nm)/Co (0.8 nm) device was ~ 3825 Ω and ~4000 Ω in normal and reversed state, respectively. The higher resistance in reversed state is due to the oxidation of the Co.

For the two devices (Pt/GdO_x and Co/GdO_x), a V_g of −6 V was applied at an elevated temperature of 100 °C. This was followed by measurements of the I-V curve at room temperature as shown in Supplementary Fig. 5. For the Pt/GdO_x device, the *I-V* curves measured after applying negative V_g for 60 and 120 mins, overlap with the initial *I-V* data. We do not observe any noticeable increase in the device resistance as shown in the inset of the figure. Since there is no net increase of the resistance of the device even after applying negative V_g for two hours at elevated temperature, it confirms that it is extremely difficult for oxygen ions to penetrate into Pt. The resistance of a Co/GdO_x device, on the other hand, increases drastically under the application of negative gate voltage at 100 °C in Supplementary Fig. 5b. The inset in the figure shows that the device resistance increases around five times after applying negative V_g for 120 mins.

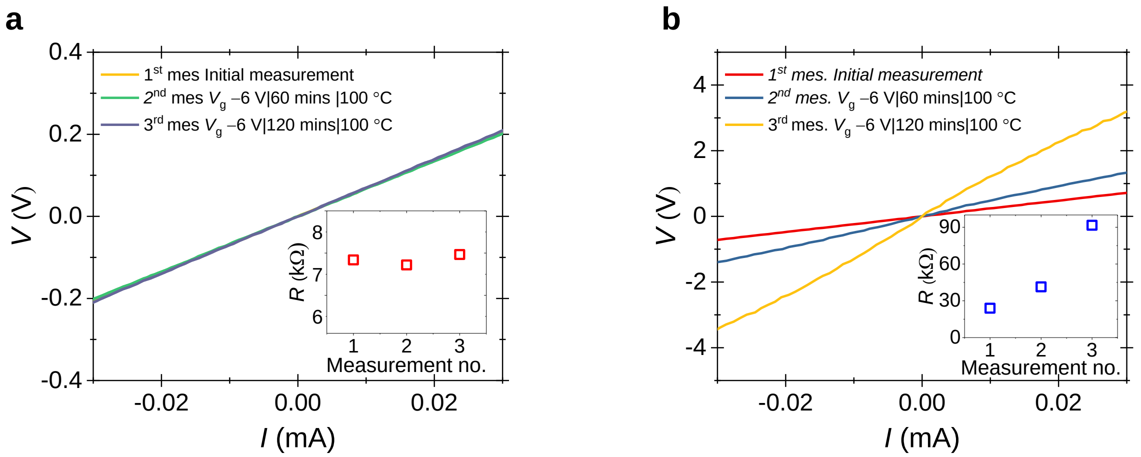

Supplementary Fig. 5. *I-V* curves after application of negative V_g. a,b, *I-V* curves for MgO/Pt (1.5 nm)/GdO_x (**a**) and MgO/Co (1 nm)/GdO_x (**b**) devices after application of negative V_g. The resistance for different measurements are shown in the insets.

Supplementary Note 5. Pt thickness dependence

The sign reversal in a Pt/Co/GdO$_x$ heterostructure is due to competition between SOTs generated by the spin Hall effect and interfacial spin-orbit coupling. If the Pt thickness increases, the spin Hall effect is expected to dominate over the interfacial torques. The Pt/Co samples with varying the Pt thickness from 1.5 to 3 nm were prepared. We find that a clear sign reversal of SOT was obtained in the samples with the Pt thickness of 2 nm and below. On the other hand, the samples with the Pt thickness of 2.5 nm and higher do not show any sign reversal in the SOT direction. Supplementary Fig. 6 shows the harmonic Hall voltage measurements for representative Pt thicknesses, t_{Pt}. It can be observed that a clear sign reversal is obtained for samples with a Pt thickness of 2 nm. For the samples with a t_{Pt} of 2.5 and 3 nm, no sign reversal is obtained even when the Co is oxidized to a very high extent (indicated by reduction in R_{AHE} to ~10 %).

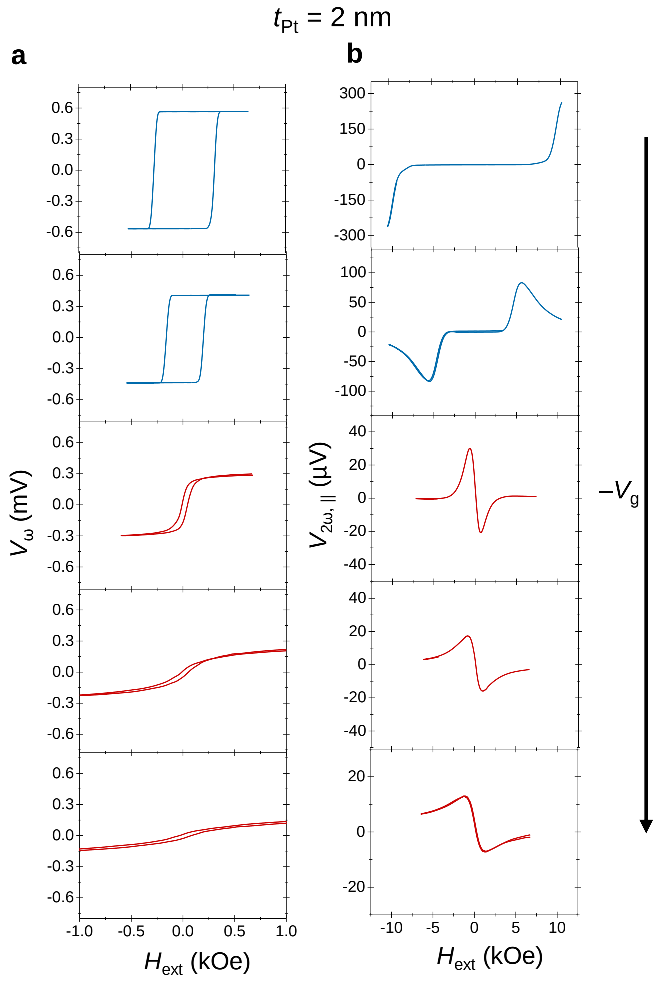

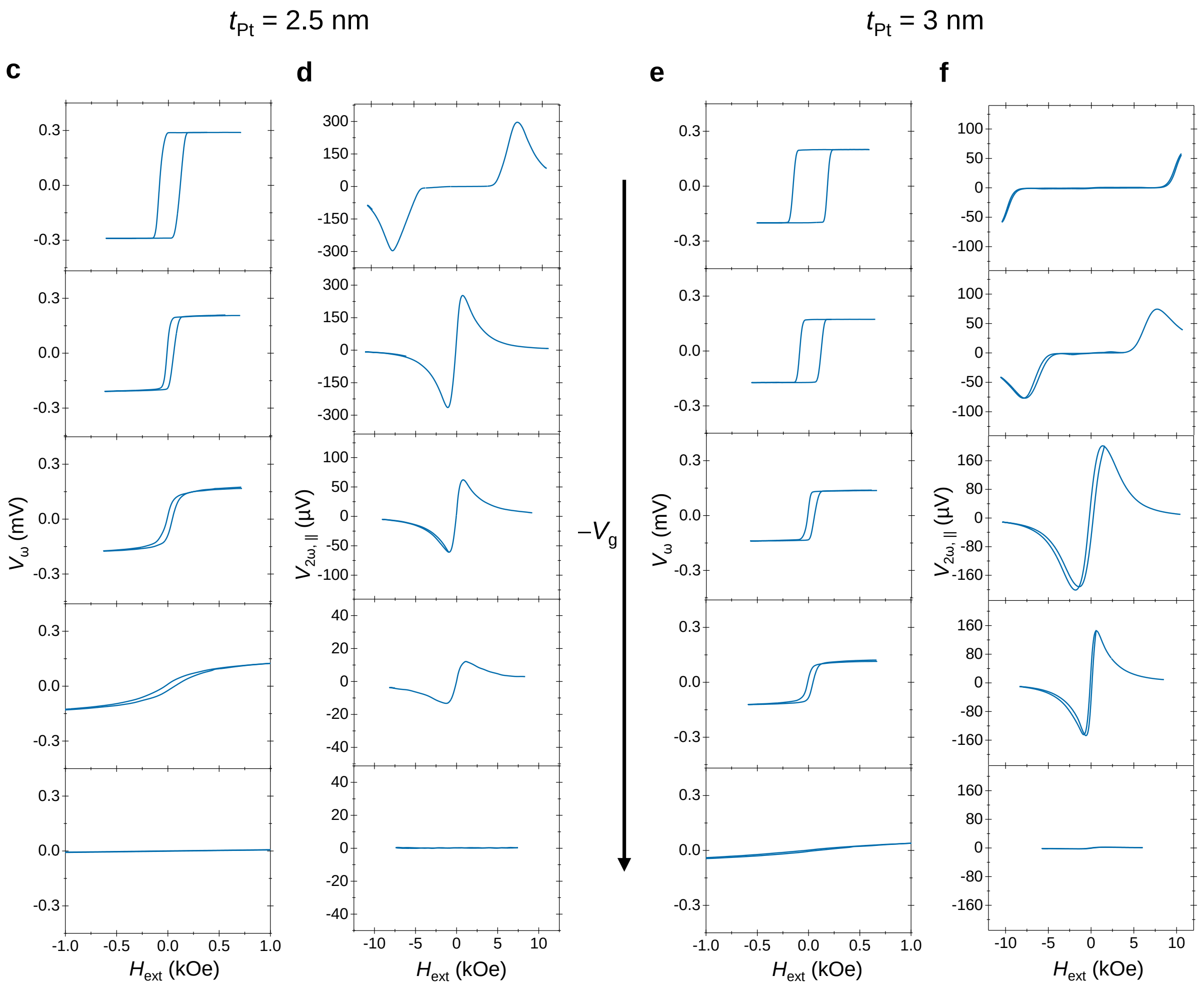

Supplementary Fig. 6. Modulation of SOTs with varying the Pt thickness. a-f, Evolution of the first (**a**,**c**,**e**) and second (**b**,**d**,**f**) harmonic signals with negative gate voltage applications for Pt (t_{Pt})/Co (0.8 nm) devices with varying the Pt thickness, t_{Pt}.

Supplementary Note 6. Effect of the Co/GdO$_x$ interface

In order to rule out that the Co/GdO$_x$ interface is a source of negative torque, we remove the Pt layer from our device. In the absence of Pt layer, any torque due to the Co/GdO$_x$ interface should be visible in the harmonic measurements. Co (1 nm)/GdO$_x$ film was deposited and fabricated with a gate oxide thickness of ~ 30 nm. Since in the absence of Pt the device has in-plane anisotropy, we use the in-plane second harmonic technique to evaluate presence of SOTs[1]. In this technique, a magnetic field is rotated in the plane of the device while passing an ac-current through it, and the subsequent first (V_ω) and second harmonic ($V_{2\omega}$) voltages are measured. Supplementary Fig. 7 shows the first and second harmonic signal obtained from a Pt (4 nm)/Co (1 nm)/TaO$_x$ device with an in-plane magnetic anisotropy (device was not annealed). The presence of SOT can be assessed from $V_{2\omega}$ which resembles a cosθ function in the presence of anti-damping torque[1].

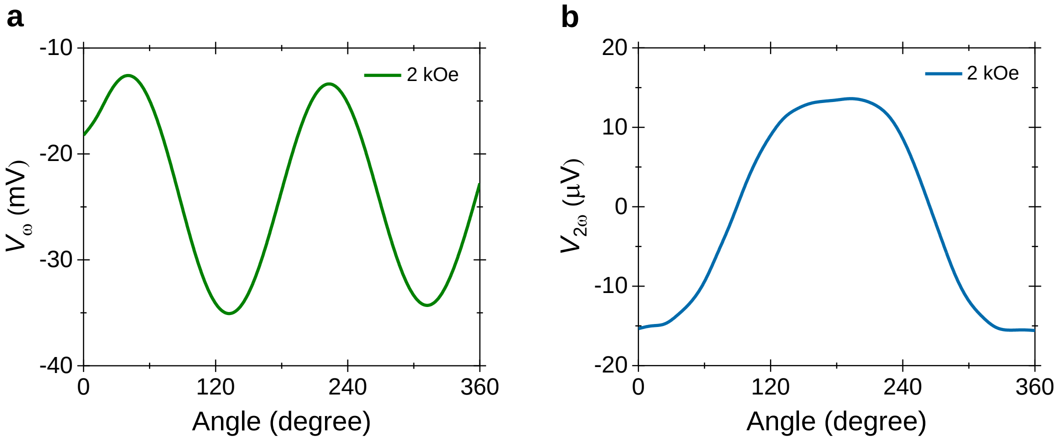

Supplementary Fig. 7. Second harmonic measurement for an in-plane SOT system. a,b, First (**a**) and second (**b**) harmonic Hall voltage for a Pt (4 nm)/Co (1 nm) device with in-plane magnetic anisotropy. Value on the x-axis represents the angle between the applied in-plane magnetic field (2 kOe) and the current. The current density through the device is 1.76×10^{10} A m^{-2}.

However, the harmonic measurements on the Co/GdO$_x$ device do not show any discernible second harmonic signal. Supplementary Fig. 8a,b shows the first and second harmonic signal from the Co/GdO$_x$ device. While the first harmonic signal is similar to that in Supplementary Fig. 7a, there is no visible second harmonic signal from this device. The $V_{2\omega}$ shown in Supplementary Fig. 8b mostly consists of the noise measured by the lock-in amplifiers. The absence of any second harmonic signal in the Co/GdO$_x$ device indicates the absence of any form of current-induced torque in the device. In addition, we apply a negative V_g on the device at an elevated temperature for an hour to ensure that Co/GdO$_x$ interface is sufficiently oxidized. The resistance of the device increases from ~ 28 kΩ to ~ 31 kΩ confirming the migration of oxygen in the Co layer. However, as shown in Supplementary Fig. 8d, the subsequent harmonic measurements also do not show any

presence of SOTs in the device. Therefore, we conclude that Co/GdO$_x$ or even over-oxidized Co/GdO$_x$ interface is not a source of any form of SOT.

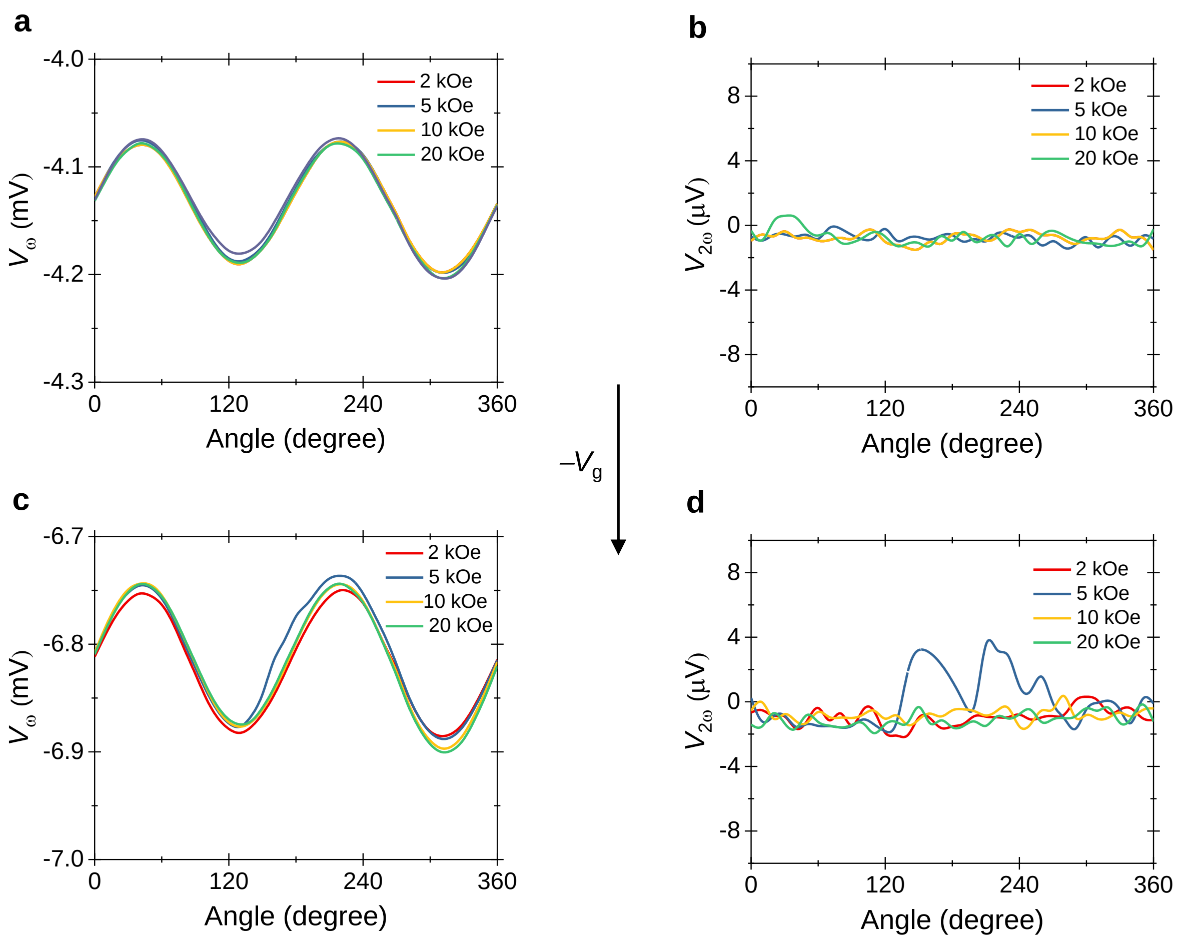

Supplementary Fig. 8. Second harmonic measurements for a Co/GdO$_x$ device. a-d, First (**a,c**) and second (**b,d**) harmonic Hall voltage for a Co (1 nm)/GdO$_x$ device with an in-plane magnetic anisotropy. The harmonic voltages are plotted as a function of angle between the applied in-plane magnetic field and the current. Before the measurements in **c,d** a negative V_g of –3.5 V was applied at 100 °C for 1 hour. The current density through the device is 1.76×10^{10} A m^{-2} for all the measurements.

Supplementary Note 7. Evaluation of effective spin Hall angle or the SOT efficiency (ξ_{SOT})

The ξ_{SOT} used in the main text can also be considered as the effective spin Hall angle which is different from the intrinsic spin Hall angle of Pt. ξ_{SOT} is equal to the intrinsic spin Hall angle for a system in which the SOTs are derived only from the pure spin Hall effect. The use of ξ_{SOT} helps in comparison between different SOT systems and in our case devices in different states. ξ_{SOT} is evaluated from the longitudinal effective field (H_L) using the equation

$$\xi_{SOT} = \frac{2eM_s t_{FM}}{\hbar J} H_L. \quad (1)$$

Here t_{FM} is the thickness of the ferromagnetic layer, J is the current density, e is the electron charge, $\hbar$ is the reduced Plank's constant, and M_s is the saturation magnetization of the ferromagnet.

On application of negative V_g in our devices, the effective thickness of the Co layer decreases due to Co oxidation. Therefore, in order to evaluate the accurate value of ξ_{SOT} for the devices, the effect of oxidation and subsequent thickness reduction is considered. The effective thickness of the Co layer is evaluated from the magnitude of V_{AHE} using the relation

$$V_{AHE} \alpha \frac{t_{Co}^2}{\rho_{Co} t_{Pt} + \rho_{Pt} t_{Co}}. \quad (2)$$

Here t_{Co} and t_{Pt} are the thickness of Co and Pt layer, respectively, and ρ_{Co} and ρ_{Pt} are their corresponding resistivity. Supplementary Fig. 9 shows the ξ_{SOT} corresponding to the H_L values shown in Fig. 3d of the main text.

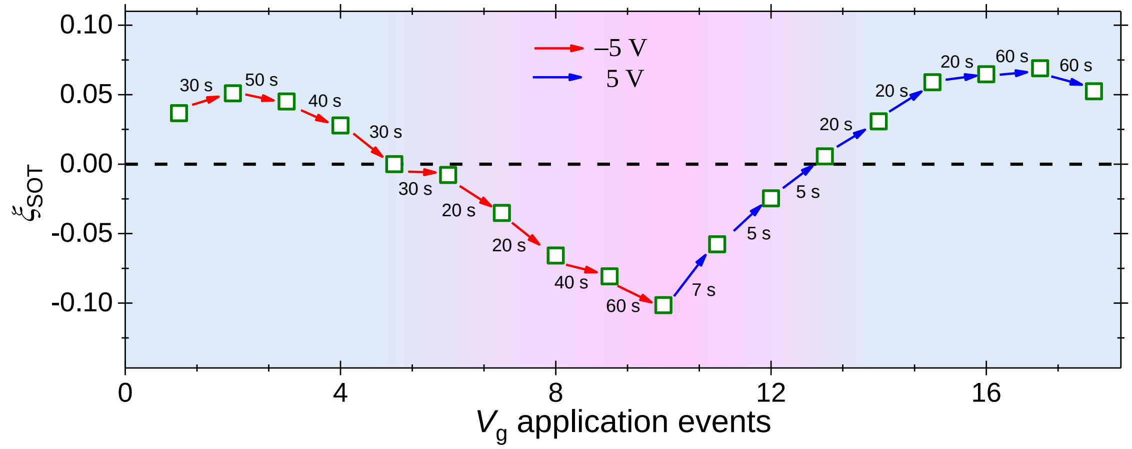

Supplementary Fig. 9. Progressive evolution of ξ_{SOT}. Evolution of ξ_{SOT} in a Pt (1.5 nm)/Co (0.8 nm) device on application of subsequent gate voltage pulses. V_g pulses were applied at room temperature while the H_L measurements were conducted at 160 K. Red arrows represent applications of negative V_g pulses while blue arrows indicate positive V_g application events. The duration of the V_g pulse is indicated adjacent to the arrows.

Supplementary Note 8. Relation between the oxygen content in Co to the device state

The amount of oxygen in the Co layer for the different device states in Fig. 3d is evaluated from the value of R_{AHE}. The oxidation of the Co results in the formation of CoO which being non-magnetic decreases the R_{AHE}.[2] The ratio of R_{AHE} for a particular device state to its maximum value (initial state without any oxygen) provides an approximate idea about the ratio of O to Co (N_O/N_{Co}) atoms in the Co layer. Supplementary Fig. 10 shows the plot of N_O/N_{Co} for the different Pt (1.5 nm)/Co (0.8 nm) device states shown in Fig. 3d of the main text. Short consecutive V_g pulses are applied to attain these device states. The color of the data point represents the measured value of H_L and thus denotes the device state. On applying negative V_g when the N_O/N_{Co} ratio exceeds ~ 0.35, the state of the device reverses *i.e.* the interfacial torque exceeds the spin Hall torque and the SOT has a negative polarity. Subsequently, when a positive V_g is applied to the device to reduce the N_O/N_{Co} ratio below 0.35, the device returns to the normal state of the positive SOT polarity.

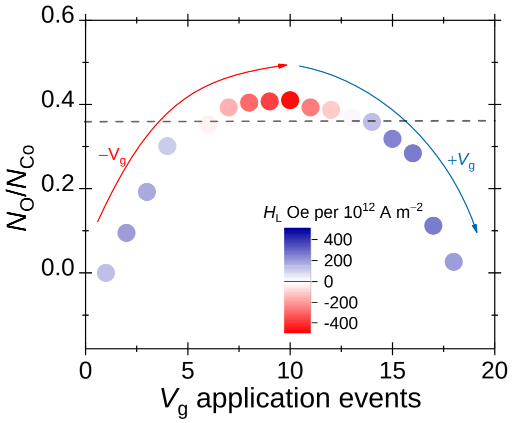

Supplementary Fig. 10. N_O/N_{Co} ratio with different voltage application events. The ratio of N_O/N_{Co} atoms in the Co layer for a Pt (1.5 nm)/Co (0.8 nm) device for the various device states. The color of the data points represents the value of longitudinal effective field, H_L. Blue and red color represents normal and reversed state of the device, respectively.

Supplementary Note 9. First principles calculations

In order to simulate the oxygen migration effect in the Co film on top of Pt, we start from a CoO/Pt bilayer. At room temperature, bulk CoO crystallizes in the rocksalt structure and is in paramagnetic phase. It has a Néel temperature of 293 K and a magnetic moment of 3.98 μ_B.[3,4] Therefore, in the first principles calculations, we consider a CoO (m)/Pt (n) slab supercell consisting of m CoO mono layers (MLs) in the rock-salt structure placed atop of n MLs of fcc Pt along the [001] direction. When there are two or more number of CoO layers in the system, we use an in-plane lattice of, $a_{\parallel} = \frac{4.05 \text{ Å}}{\sqrt{2}} = 2.86$ Å, which is between that of bulk rocksalt CoO (4.26 Å) and of bulk Pt (3.92 Å), otherwise we use $a_{\parallel} = \frac{3.92 \text{ Å}}{\sqrt{2}} = 2.77$ Å. The removal of m–N_O oxygen atoms (one per layer) from the CoO film results in CoO (N_O)/Co (m–N_O)/Pt (n) slab trilayer. We find that the average relaxed interlayer distance in the Co thin film is about 1.35 Å$\left(\approx \frac{a_{\parallel}}{2}\right)$, indicating the formation of a strained Co film in bcc lattice structure. Bulk bcc Co (with 2.8 Å lattice constant and magnetic moment of 1.53 μ_B) is a metastable structure that has been experimentally observed[5]. The supercell slab shown in Fig. 4a of the main text denotes the CoO (5 ML)/Co (5 ML)/Pt (4 ML) trilayer as a representative example. From first principles calculation we find a magnetic moment of 0.25 μ_B for proximity induced magnetic moment on the interfacial Pt layer, 1.7 μ_B for Co atoms in the bcc Co film and 2.7 μ_B for Co atoms in the rocksalt CoO film, consistent with the previous theoretical calculations[6-10]. The difference between the experimental magnetic moment and the theoretical spin moment yields an estimated value of 1.3 μ_B for the orbital moment of CoO, which is 5 times larger than the theoretical value, 0.25 μ_B[11].

Using the tight-binding (TB) Hamiltonian ($\hat{H}_{\vec{k}}$), obtained from the VASP-Wannier90 calculations[12] as detailed in ref. 13, we have calculated the SOT versus magnetization orientation from[14]

$$\vec{\boldsymbol{\tau}}_{\mathrm{FL}} = \frac{1}{\pi M_s N_k} \vec{\mathbf{m}} \times \sum_{\vec{\mathbf{k}}} \mathbf{Tr}\left[\frac{\partial \hat{\mathbf{H}}_{\vec{\mathbf{k}}}}{\partial \vec{\mathbf{m}}} \mathbf{Im}\left(\hat{\mathbf{G}}_{\vec{\mathbf{k}}}\right) \frac{\partial \hat{\mathbf{H}}_{\vec{\mathbf{k}}}}{\partial \mathbf{k_x}} \mathbf{Im}\left(\hat{\mathbf{G}}_{\vec{\mathbf{k}}}\right)\right] \quad (3)$$

$$\vec{\boldsymbol{\tau}}_{\mathrm{DL}} = \frac{2}{M_s N_k} \vec{\mathbf{m}} \times \sum_{\mathbf{mn}\vec{\mathbf{k}}} \mathbf{Re}\left[\frac{\mathbf{Im}\left(\left[\partial \hat{\mathbf{H}}_{\vec{\mathbf{k}}}/\partial \vec{\mathbf{m}}\right]_{\mathbf{mn}} \left[\partial \hat{\mathbf{H}}_{\vec{\mathbf{k}}}/\partial \mathbf{k_x}\right]_{\mathbf{nm}}\right)}{(\boldsymbol{\varepsilon}_{\mathbf{n}\vec{\mathbf{k}}} - \boldsymbol{\varepsilon}_{\mathbf{m}\vec{\mathbf{k}}} - \mathbf{i}\boldsymbol{\eta})^2}\right]. \quad (4)$$

Here, $\hat{\mathbf{G}}_{\vec{\mathbf{k}}} = 1/(E_{\mathrm{F}} - \hat{\mathbf{H}}_{\vec{\mathbf{k}}} - i\eta)$ is the Greens function calculated at the Fermi energy, E_{F},$[\ldots]_{\mathrm{mn}}$ is the matrix element in the basis set of the eigenstates of the Hamiltonian, M_s is the total magnetic moment of the Co layer, $\vec{\mathbf{m}}$ is the unit vector along the magnetization direction, $\varepsilon_{\mathrm{n}\vec{\mathrm{k}}}$ are the Hamiltonian eigenvalues, $\eta = \hbar/2\tau$ is the energy broadening value which is inversely proportional to the electronic relaxation time τ, and N_{k} =120×120×1 is the number of k-point mesh for the Brillouin-zone sampling. Having determined the SOTs for a set of magnetization

orientations, we fit the results to the $\vec{\boldsymbol{\tau}}_{\mathbf{FL}} = \tau_{FL}^0 \vec{\mathbf{m}} \times \vec{\mathbf{y}}$ and $\vec{\boldsymbol{\tau}}_{\mathbf{DL}} = \tau_{\mathrm{DL}}^0 \vec{\mathbf{m}} \times (\vec{\mathbf{m}} \times \vec{\mathbf{y}})$ expressions which in turn yield the SOT coefficients, τ_{FL}^0 and τ_{DL}^0, respectively.

Supplementary Fig. 11a shows the calculated SOT coefficients, $\tau_{\mathrm{FL/DL}}^0$ versus the Pt thickness for the Pt ($N_{\text{Pt-ML}}$ ML)/Co (10 ML) bilayer system and $\eta = 0.1$ eV. The TB Hamiltonian for the Pt ($N_{\text{Pt-ML}}$)/Co (10 ML) bilayer is determined from the Pt (16 ML)/Co (10 ML) supercell TB Hamiltonian through sequential removal of the surface Pt atomic orbitals. The dashed curve denotes the fit of the damping like-SOT (DL-SOT) coefficient to the spin-diffusion model, $e\tau_{\mathrm{DL}}^0 = \frac{A\sigma_{\mathrm{SH}}}{M_{\mathrm{s}}}\left(1 - \mathrm{sech}\left(\frac{t_{\mathrm{Pt}}}{\lambda_{\mathrm{s}}}\right)\right)$ which yields a spin diffusion length of $\lambda_{\mathrm{S}} = 0.8$ nm and an effective spin Hall conductivity of $\sigma_{\mathrm{SH}} = 2 \times 10^5 [\frac{\hbar}{2\mathrm{e}}](\Omega^{-1}\ \mathrm{m}^{-1})$. On the other hand, the field like-SOT (FL-SOT) is relatively independent of the Pt thickness for relatively thick Pt.

The effective Rashba coefficient (ERC) for a bilayer can be determined either from the shift of the spin resolved expectation value of the $\vec{\boldsymbol{k}}$ vector on the Fermi surface for an in-plane magnetization direction[15], or using the FL-SOT expression, $K_0\tau_{\mathrm{FL}}^0 = P\alpha_{\mathrm{R}}\sigma_{\mathrm{xx}}/M_{\mathrm{s}}$[16], for a 2D Rashba electron gas, where $K_0 = \hbar^2/2m_{\mathrm{e}} = 3.8$ eV Å^2, $P \approx \frac{D_\uparrow(E_{\mathrm{F}}) - D_\downarrow(\mathrm{E_F})}{D_\uparrow(E_{\mathrm{F}}) + D_\downarrow(E_{\mathrm{F}})}$ is the interfacial spin polarization factor, $D_\sigma(E_{\mathrm{F}})$ is the spin resolved density of states at the Fermi energy (E_{F}), m_{e} is the free electron's mass, and σ_{xx} is the longitudinal conductivity which is calculated from

$$\sigma_{\mathrm{xx}} = \frac{1}{\pi N_{\mathrm{k}}} \sum_{\vec{\mathrm{k}}} Tr\left[\frac{\partial \hat{H}_{\vec{\mathrm{k}}}}{\partial k_{\mathrm{x}}} Im\left(\hat{G}_{\vec{\mathrm{k}}}\right) \frac{\partial \hat{H}_{\vec{\mathrm{k}}}}{\partial k_{\mathrm{x}}} Im\left(\hat{G}_{\vec{\mathrm{k}}}\right)\right]. \tag{5}$$

It should be mentioned that for simplicity the prefactor $\mathrm{e}^2/V\hbar$ (V is the volume of the active region) is not included in the above expression for the conductivity, therefore the units of σ_{xx} in Supplementary Equation 5 is Å^2. In this work, we use the latter approach to calculate the ERC, defined as $P\alpha_{\mathrm{R}}$ which allows for a quantitative comparison with the experimental measurements.

In Supplementary Fig. 11b, we display the variation of the effective spin Hall conductivity (left-hand ordinate) and the effective Rashba coefficient (right-hand ordinate) as a function of Co thickness for the Pt (4 ML)/Co ($N_{\text{Co-ML}}$) bilayer where we considered the spin diffusion constant λ_{s}= 0.8 nm TB Hamiltonian. The result shows that for $N_{\text{Co-ML}} > 2$ the effective σ_{SH} is relatively independent of Co thickness and close to the value of $2.2\times10^5 [\frac{\hbar}{2\mathrm{e}}](\Omega^{-1}\ \mathrm{m}^{-1})$ for bulk Pt[12]. On the other hand, the ERC depends strongly on the Co thickness in the ultrathin film limit and exhibits a sign reversal around 4 MLs of Co. However, the sign reversal of the SOT on incorporation of oxygen is not due to the reduction of the Co thickness as shown in Fig. 4b of the main text.

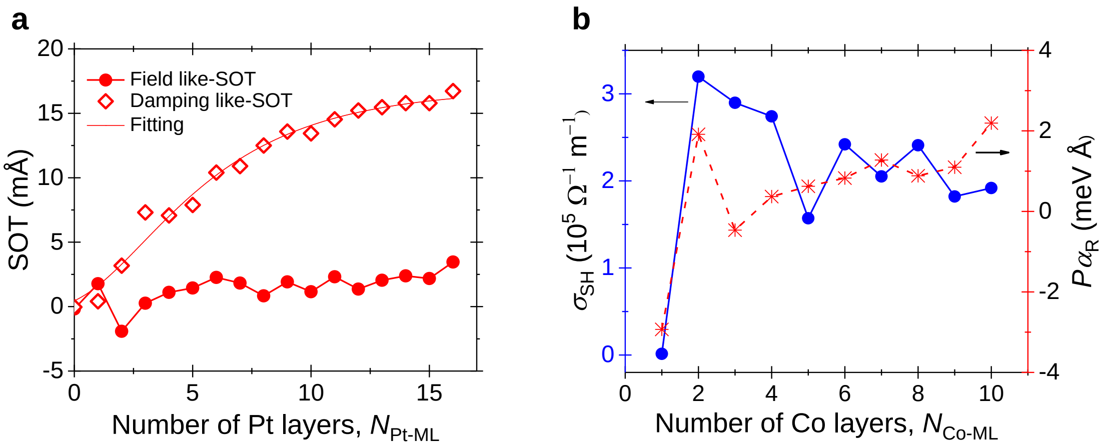

Supplementary Fig. 11. First principle calculation results. a, DL and FL spin orbit torque versus the number of Pt layers for Pt ($N_{\text{Pt-ML}}$ ML)/Co (10 ML) bilayer without oxygen for $\eta = 0.1$ eV. The dashed line denotes the fit of the *ab initio* results to the spin diffusion model, $e\tau^0_{\text{DL}} = \frac{A\sigma_{\text{SH}}}{M_s}\left(1 - \text{sech}\left(\frac{t_{\text{Pt}}}{\lambda_s}\right)\right)$, where $A = 7.68$ Å^2 is the area of the unit cell, $M_s = 18.6\mu_B$, is the total magnetic moment per unit cell, and σ_{SH} is the spin Hall conductivity of bulk Pt. The fit yields $\sigma_{\text{SH}} = 0.04$ eÅ^{-1} $\equiv 2 \times 10^5 \left[\frac{\hbar}{2e}\right]\left(\Omega^{-1}\ \text{m}^{-1}\right)$ for the effective spin-Hall conductivity and $\lambda_s = 0.8$ nm for the spin diffusion length of Pt. **b**, Spin Hall conductivity (left-hand ordinate) and effective Rashba SOC strength (right hand ordinate) versus Co thickness for the Pt (4 ML)/Co ($N_{\text{Co-ML}}$ ML) bilayer.

Supplementary Note 10. Device stability

In order to check the stability of the normal and reversed state of the device, we continuously monitor the second harmonic SOT signal with $H_{ext} \parallel I_{ac}$ for a Pt (2 nm)/Co (0.8 nm) device over a period of 12 hours at room temperature for both normal and reversed state. Supplementary Fig. 12a,b show the measured second harmonic signal for the normal and reversed device state. There is no noticeable degradation in the measure signal which signifies that the device is quite stable at room temperature in both the normal and the reversed state.

In a separate experiment, we also monitor the device resistance for both the normal and reversed state. If the devices are unstable, there would be migration of oxygen to or from the Co layer which would result in a decrease or increase of the device resistance. However, we do not notice any observable change in the device resistance as shown in Supplementary Fig. 12c. The resistance versus time curve could not be fit with an exponential decay function of reasonable long relaxation time (e.g. ~ 10 years). This confirms that the position of oxygen in the device and subsequently the device state is quite stable for an extended period of time.

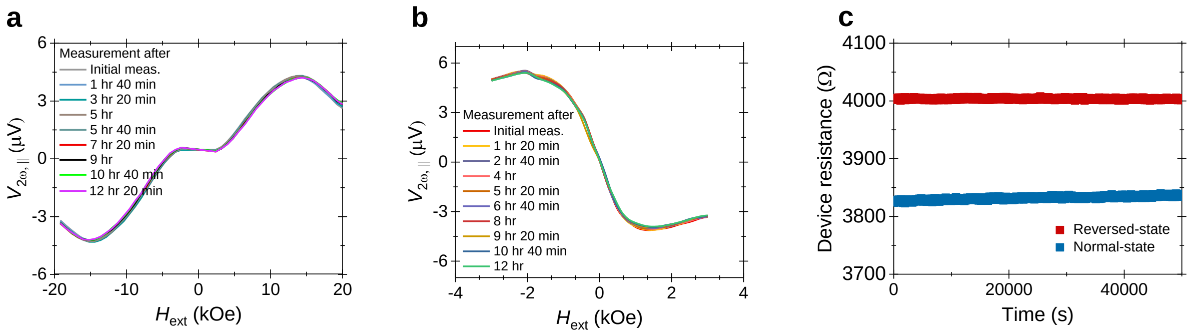

Supplementary Fig. 12. Stability of normal and reversed device. a,**b**, Continuous monitoring of the second harmonic signal at room temperature with $H_{ext} \parallel I_{ac}$ for a Pt (2 nm)/Co (0.8 nm) device in normal state (**a**) and reversed state (**b**). **c**, Resistance of the device over an extended period of time for the device in the normal and reversed state.

Supplementary references

1 Avci, C. O. *et al.* Interplay of spin-orbit torque and thermoelectric effects in ferromagnet/normal-metal bilayers. *Phys. Rev. B* **90**, 224427 (2014).

2 Bi, C. *et al.* Reversible Control of Co Magnetism by Voltage-Induced Oxidation. *Phys. Rev. Lett.* **113**, 267202 (2014).

3 Roth, W. L. Magnetic Structures of MnO, FeO, CoO, and NiO. *Phys. Rev.* **110**, 1333-1341 (1958).

4 Jauch, W., Reehuis, M., Bleif, H. J., Kubanek, F. & Pattison, P. Crystallographic symmetry and magnetic structure of CoO. *Phys. Rev. B* **64**, 052102 (2001).

5 Prinz, G. A. Stabilization of bcc Co via Epitaxial Growth on GaAs. *Phys. Rev. Lett.* **54**, 1051-1054 (1985).

6 Wdowik, U. D. & Parlinski, K. Lattice dynamics of CoO from first principles. *Phys. Rev. B* **75**, 104306 (2007).

7 Feng, X. Electronic structure of MnO and CoO from the B3LYP hybrid density functional method. *Phys. Rev. B* **69**, 155107 (2004).

8 Solovyev, I. V., Liechtenstein, A. I. & Terakura, K. Is Hund's Second Rule Responsible for the Orbital Magnetism in Solids? *Phys. Rev. Lett.* **80**, 5758-5761 (1998).

9 Wei, P. & Qi, Z. Q. Insulating gap in the transition-metal oxides: A calculation using the local-spin-density approximation with the on-site Coulomb U correlation correction. *Phys. Rev. B* **49**, 10864-10868 (1994).

10 Anisimov, V. I., Zaanen, J. & Andersen, O. K. Band theory and Mott insulators: Hubbard U instead of Stoner I. *Phys. Rev. B* **44**, 943-954 (1991).

11 Schron, A. *et al.* Crystalline and magnetic anisotropy of the 3d-transition metal monoxides MnO, FeO, CoO, and NiO. *Phys. Rev. B* **86**, 115134 (2012).

12 Mostofi, A. A. *et al.* Anupdated version of wannier90: A tool for obtaining maximally-localised Wannier functions, *Comput. Phys. Commun.***185**, 2309 (2014)

13 Mahfouzi, F., Kim, J. & Kioussis, N. Intrinsic damping phenomena from quantum to classical magnets: An ab initio study of Gilbert damping in a Pt/Co bilayer. *Phys. Rev. B* **96**, 214421 (2017).

14 Mahfouzi, F. & Kioussis, N. First-principles study of the angular dependence of the spin-orbit torque in Pt/Co and Pd/Co bilayers. *Phys. Rev. B* **97**, 224426 (2018).

15 Haney, P. M., Lee, H.-W., Lee, K.-J., Manchon, A. & Stiles, M. D. Current-induced torques and interfacial spin-orbit coupling. *Phys. Rev. B* **88**, 214417 (2013).

16 Manchon, A. & Zhang, S. Theory of spin torque due to spin-orbit coupling. *Phys. Rev. B* **79**, 094422 (2009).
